# Special Issue: Ecology of Sex and Sexual Communication in Insects

**DOI:** 10.3390/insects12020137

**Published:** 2021-02-05

**Authors:** Emily R. Burdfield-Steel, Ally Rachel Harari

**Affiliations:** 1 Institute for Biodiversity and Ecosystem Dynamics, University of Amsterdam, 1098 XH Amsterdam, The Netherlands; 2 Department of Entomology, Agricultural Research Organization, The Volcani Center, Bet Dagan 50250, Israel

Sexual reproduction places constraints on both the place and time in which individuals can reproduce, as the sperm and ova need to meet in a certain location within a specific time frame for successful reproduction. Ecology, as the interplay between natural and sexual selection and the environment strongly affects the patterns of sexual behavior that we see in nature. Individuals must adapt to both their environment, and the community they share it with, in order to maximize their chances of reproduction. The diversity of mating systems and sexual communication modes in insects across taxa is intriguing, and in many species, even small mismatches in timing or the mode of information transfer can result in a failure to mate, while overlap in these traits between species may lead to costly interspecific mating mistakes. In this editorial, we focus on the effect of ecology on sexual behavior and communication in insects, and highlight the importance of understanding both, particularly in light of increasing anthropogenic changes and their dramatic effects on insect populations.

## 1. Ecology of Sex

Ecological factors determine patterns of sexual communication and behavior. Environmental characteristics affect the mode of sexual communication; noisy environments are not expected to promote the evolution of acoustic communication, while visual communication may not be adaptive in nocturnal or cave dwelling insects. Resource availability is known to affect mate defending or monopolizing and the occurrence and different levels of polyandry, monandry or polygyny in a population. Food availability or its scarcity affect female distribution and therefore sex ratio and population density, thus influence the degree of intra male and sperm competition, and sexual harassment may lead to a conflict of interest between the sexes.

## 2. Sexual Communication

Communication is vital both for the location and selection of a suitable mate. Insects represent a huge range of sexual communication modes, spanning chemical, visual, acoustic and vibratory. Insects represent one of the best-studied groups for long-distance sexual communication, using species-specific sex pheromones. The high specificity of sex pheromones may evolve as a means to avoid wasting energy and materials while mating with the wrong species, but the variation in the pheromone characteristics within a population may be used to select a better mate. Sex pheromones are often synergized with the plant volatiles, thus, in addition to closing the time gap between mating and oviposition, it allows the females to locate males and oviposition sites at the same time or assist males in finding receptive females.

## 3. Sexual Communication and Agriculture

Long-range pheromone signaling was first described in the Lepidoptera, and has become the basis of a multi-million agricultural industry worldwide. Synthesized sex pheromones are commonly used to attract males into pheromone-baited traps, as a tool to monitor pest populations, and to time interventions. Aggregation pheromones are used to monitor and to attract and kill pest insects, and the female sex pheromone of many moth and some scale insect species is used for mating disruption, preventing males from locating receptive females. These environmentally friendly methods allow for the control of many insect pests, without the costs of environmental destruction associated with pesticides.

## 4. Ecology of Communication

One thing that is clear from the success of mating disruption as a management intervention is the critical role the environmental conditions play on sexual communication. While human-produced chemical and pheromones are a relatively new phenomena, species have long had to adapt to interference from closely related species, and this character displacement is thought to have created the huge variation seen in many modes of communication. Beyond reproductive interference, communication on the one hand, is shaped by the species ecology (for example their densities and dispersal), and, on the other, may influence their ability to expand into new environments. Long-range pheromone attraction, for example, can allow species to successfully find mates, even when at low frequencies, while signals like cuticular hydrocarbons, which require much closer proximity, can be used to recognize close kin or previous mates.

## 5. Ecology of Decision Making

As well as affecting the transmission of signals, ecology also influences the social landscape, through the competition for mates, the risk of mating with close kin, male harassment of females, and the distribution of resources among potential mates. Hence, affecting sexual selection and the information required to make mating decisions. Griffin and Symonds (available as an early bird in this Special Issue) demonstrate this in a rare example of a polygynous harem-forming insect [1]. They found that the number of females in male harems of the polygynous bark beetle Ips grandicollis was significantly affected by the resource availability in the surrounding habitat. When resources, and hence population density, was higher, the average number of females per male increased. This was true despite prior evidence that female finesses is negatively affected by larger harem sizes, suggesting that resource availability can impact male and female fitness in different ways.

## 6. The Importance of the Topic

As insects continue to suffer significant losses in the face of human-induced climatic and environmental change, understanding the full role of ecology in governing and shaping their sexual selection and communication becomes ever more urgent. In the work of Griffin and Symonds, for example, the biggest population densities, and hence harem sizes, ever found in harvested pine plantations, when huge numbers of trees are felled, creating an abundance of fresh wood debris for the insects to feed on. There is a lack of evidence that females adapt their mate choice and distribution to these high densities and that females in large harems optimize the space between their breeding chambers to reduce competition with other females; this may indicate that such scenarios were not commonplace prior to human management of forests.

Human activities affect not just the environmental conditions that shape the sexual selection and mating systems within insects, but also every mode through which sexual communication takes place. Light pollution affects visual signals, noise pollution auditory and vibrational ones. Air pollution, in particular, is expected to significantly impact insects that rely on long-distance pheromones signaling, however investigations into its effects in ecologically relevant settings have only just begun. In light of all of this, we urge researchers to focus on all aspects of insects’ biology in this rapidly emerging new world, and we believe that sex and sexual communication are paramount among these.
